# Factors associated with body weight gain and insulin-resistance: a longitudinal study

**DOI:** 10.1038/s41387-024-00283-5

**Published:** 2024-04-22

**Authors:** Carola Buscemi, Cristiana Randazzo, Anna Maria Barile, Simona Bo, Valentina Ponzo, Rosalia Caldarella, Alexis Elias Malavazos, Roberta Caruso, Piero Colombrita, Martina Lombardo, Silvio Buscemi

**Affiliations:** 1 Unit of Internal Medicine, “V. Cervello Hospital”, Palermo, Italy; 2 Dipartimento di Promozione della Salute, Materno-Infantile, Medicina Interna e Specialistica di Eccellenza (PROMISE), University of Palermo, Palermo, Italy; 3 Unit of Clinical Nutrition, Obesity and Metabolic Diseases; AOU Policlinico “P. Giaccone”, Palermo, Italy; 4 Department of Medical Sciences, University of Torino, Torino, Italy; 5 Unit of Laboratory Medicine, AOU Policlinico “P. Giaccone”, Palermo, Italy; 6 Endocrinology Unit, Clinical Nutrition and Cardiovascular Prevention Service, IRCCS Policlinico San Donato, San Donato Milanese, Italy; 7 Department of Biomedical, Surgical and Dental Sciences, University of Milan, Milan, Italy

**Keywords:** Obesity, Epidemiology

## Abstract

**Background:**

Obesity is the result of energy intake (EI) chronically exceeding energy expenditure. However, the potential metabolic factors, including insulin resistance, remain unclear. This study longitudinally investigated factors associated with changes in body weight.

**Subjects:**

A cohort of 707 adults without diabetes were investigated at the 4-year follow-up visit. The habitual intake of energy and macronutrients during the past 12 months was assessed using a validated Food Frequency Questionnaire for the local population. Homeostatic model assessment of β-cell function and insulin resistance (HOMA-IR) was used as a surrogate measure of insulin resistance. Additionally, *PNPLA3* was genotyped.

**Results:**

Eighty-seven participants were weight gainers (G; cutoff value = 5 kg), and 620 were non-gainers (NG). Initial anthropometric (G vs. NG: age, 44 ± 13 vs 51 ± 13 years, *P* < 0.001; body mass index, 27.8 ± 6.5 vs 28.1 ± 5.1 kg/m^2^, *P* = ns; body weight, 76.7 ± 22.1 vs 74.2 ± 14.7 kg, *P* = ns; final body weight, 86.3 ± 23.7 vs 72.9 ± 14.2 kg, *P* < 0.001) and diet characteristics, as well as insulin concentrations and HOMA-IR values, were similar in both groups. Four years later, G showed significantly increased EI, insulin concentrations, and HOMA-IR values. G had a higher prevalence of the PNPLA3 CG and GG alleles than NG (*P* < 0.05). The presence of G was independently associated with age (OR = 1.031), EI change (OR = 2.257), and unfavorable alleles of PNPLA3 gene (OR = 1.700). Final body mass index, waist circumference, and EI were independently associated with final HOMA-IR (*P* < 0.001).

**Conclusions:**

EI is associated with body weight gain, and genetic factors may influence the energy balance. Insulin resistance is a consequence of weight gain, suggesting a possible intracellular protective mechanism against substrate overflow.

**Clinical trial registration:**

ISRCTN15840340.

## Introduction

Obesity is an increasingly widespread condition worldwide, with an actual prevalence of over 800 million adults and a predicted prevalence of 1.5 billion people by 2035 [1]. Although it is commonly accepted that obesity is the result of an unhealthy lifestyle, with energy intake chronically exceeding energy expenditure, the roles of other potentially contributing metabolic factors remain clarified [2]. An extensively investigated but still unresolved question concerns the possibility that insulin resistance and hyperinsulinemia, in addition to being caused by obesity, primarily contribute to the development of obesity itself [3–5]. In fact, as insulin induces liposynthesis and influences appetite and energy expenditure, it was also proposed that hyperinsulinemia and insulin resistance may primarily induce weight gain [6]. However, clinical epidemiological studies in adult cohorts are inconclusive about this possibility [7, 8]. In addition, studies involving pediatric cohorts have shown contradictory results. In a longitudinal study involving children, Sedaka et al. [9] failed to observe a predictive relationship between insulin, the homeostatic model assessment of β-cell function and insulin resistance (HOMA-IR), and body weight gain. However, a similar study by Labayen et al. [10] demonstrated that insulin resistance in childhood may predict subsequent total and central adiposity gains during adolescence. Insulin resistance associated with obesity is also a common basis of metabolic Syndrome, a clinical condition to which much attention is paid, as it can evolve with the development of type 2 diabetes, hypertension, hypertriglyceridemia, low serum concentrations of high-density lipoprotein-cholesterol (HDL-C), liver steatosis, and atherosclerosis. Therefore, insulin resistance is often considered a clinical problem to address not only favorable lifestyle changes but also pharmacological treatments, as biguanides are the most commonly used drugs [11]. Therefore, ascertaining the longitudinal direction of the association between insulin resistance/hyperinsulinemia and obesity is important because it may contribute to addressing more appropriate strategies, such as prevention-as-treatment, to counteract obesity and its complications.

In this study, we aimed to longitudinally investigate the factors associated with a 4-year body weight change, including serum insulin concentration and HOMA-IR, as a surrogate measure of insulin resistance, in a comprehensive general population cohort.

## Subjects and methods

### Participants

The Nutrition, Cardiovascular Wellness, and Diabetes (ABCD) Project (ISRCTN15840340) is a longitudinal observational single-center study of a cohort representative of the general population living in Palermo, the largest city in Sicily (Italy), with 674,742 inhabitants in 2011. The ABCD_1 study cohort was recruited in 2011 as previously described [12]. The inclusion criteria were participants aged >18 years and residing in Palermo. The demographic characteristics of the ABCD cohort were similar to, if not overlapping with, those of the general population of the same age range (18–90 years) as presented elsewhere [13]. At the conclusion of their participation in the ABCD_1 study, all participants were interviewed briefly by one of the investigators. During this interview, they received a written report detailing the results of the investigations conducted. This report also highlighted any identified clinical issues and health risk factors. An analysis of eating habits was conducted, and recommendations were provided to improve one’s dietary choices and potentially increase levels of physical activity. The report, along with its associated recommendations, was forwarded to the participants’ doctors. The original cohort was recontacted (telephone, e-mail, letter) in 2015, and those who agreed to participate in the study were re-examined between March 21 and July 31 at the Metabolism and Clinical Nutrition Laboratory of the Department of Internal Medicine at the University of Palermo. Demographic characteristics of the ABCD_2 cohort were not significantly different from those of the ABCD_1 cohort, as reported previously [14]. For this study, individuals with a known diabetes diagnosis were excluded from calculations.

The institutional Ethics Committee (“Palermo 1,” Policlinico “P. Giaccone” University Hospital, 11/03/2014, ref: 3/2015) approved the study protocol, and each participant signed an approved informed consent form.

The habitual intake of energy and macronutrients during the past 12 months was assessed using a previously validated medium-length Food Frequency Questionnaire for the local population as described elsewhere [15]. Adherence to the Mediterranean Diet was assessed by using the MEDI-LITE questionnaire [16].

Habitual physical activity level was investigated using a specific questionnaire for the local population that describes 4 levels of physical activity progressively increasing from 1 to 4 [14].

Participants underwent blood sampling for the assessment of blood chemistry and hormonal parameters. For each participant, a blood sample was frozen and stored at −80 °C for subsequent measurements. Type 2 diabetes and pre-diabetes were defined according to the most recent consensus statements [17].

### Measurements

Height and body weight were measured with the participants lightly dressed and without shoes (SECA; Birmingham, UK). Body mass index (BMI) was calculated as body weight (kg)/height^2^ (m^2^). Participants with a BMI <25 kg/m^2^ were categorized as normal-weight, while those with a BMI ≥25 kg/m^2^ were classified as overweight/obese [18]. Body circumference was measured at the umbilicus (waist circumference) and at the most prominent buttock level (hip circumference) and was used as an indirect index of body fat distribution. Systolic and diastolic arterial blood pressure (two measurements obtained at 5-min intervals in a seated position) and heart rate (Omron M6; Omron Healthcare Co., Matsusaka, Mie, Japan) were measured by physicians or dietitians, according to standardized procedures. Body composition in terms of fat mass (FM) and fat-free mass (FFM) was estimated using Bioelectrical Impedance Analysis (BIA; BIA-101 Anniversary; Akern, Firenze, Italy) following the manufacturer’s equations, as previously described [19].

### Laboratory analysis

Fasting plasma glucose, total cholesterol, HDL-C, triglyceride, uric acid, and creatinine levels were measured using standard clinical chemistry methods (Glucosio HK UV; Colesterolo totale Mod P/D; Colesterolo HDL gen 3 mod P/917; Trigliceridi; Acido urico MOD P/917; Creatinina enzimatica; Roche Diagnostics, Monza, Italy). Basal insulin concentrations (Elecsys insulina; Roche Diagnostics; Monza, Italy), high-sensitivity C-reactive protein (hs-CRP; B-analyst hs-CRP; Menarini Diagnostics; Florence, Italy), and glycated hemoglobin (HbA_1_c; B-analyst HbA1c; Menarini Diagnostics; Florence, Italy) levels were also measured. Low-density lipoprotein-cholesterol serum concentration was calculated using Friedewald’s formula [20]; glomerular filtration rate (eGFR) was estimated based on the CKD-EPI equation [21], and HOMA-IR was calculated as described by Matthews et al. [22]. DNA was purified using a QIAmp blood Mini Kit (Qiagen, Hilden, Germany), and DNA samples were quantified using spectrophotometry. Genotyping for patatin-like phospholipase domain containing 3 (PNPLA3) (rs738409) and transmembrane 6 superfamily 2 (TM6SF2) (rs58542926) was performed using the TaqMan SNP genotyping allelic discrimination method (Applied Biosystems, Foster City, CA, USA).

### Statistical analysis

Data are reported as the means ± SD for continuous variables and as percentages for categorical variables. A body weight increase of <3 kg in 10 years is commonly considered physiological in adults [23]. Therefore, based on the body weight change from 2011 to 2015, an arbitrary cutoff value of 5 kg was utilized to distinguish individuals who gained weight (*weight gainers*, G) from those who did not gain weight (*weight non-gainers*, NG). Furthermore, it was demonstrated that a body weight gain of 5 kg had a significant impact on different aspects of health [24]. The Student’s *t* test for unpaired data was used to compare continuous variables between groups. The changes in variables between ABCD_1 and ABCD_2 were assessed using paired sample Student’s *t* tests. Differences in categorical variables were analyzed using the *χ*^2^ test. Pearson’s correlation coefficients were calculated to explore the associations between continuous variables. The absolute differences (Δ = value measured in ABCD_2 – value measured in ABCD_1) between the continuous variables of interest were categorized as 0 (Δ≤0) or 1 (Δ > 0). Multiple logistic binary regression analysis was used to predict the outcomes (body weight change and HOMA-I). Statistical significance was set at *P* value < 0.05. All analyses were performed using the Systat software (Windows version 13.0; San Jose, CA, USA).

## Results

**Fig. 1:**
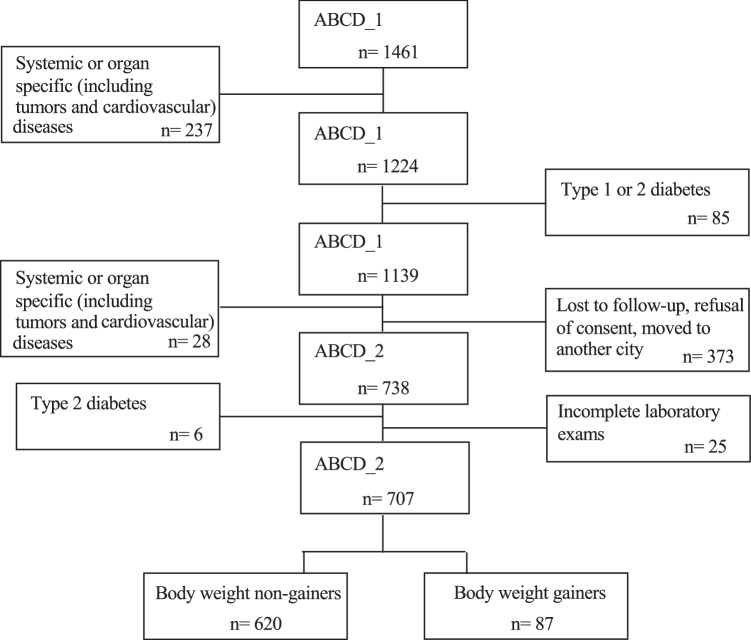
Flow chart of participant selection.

A total of 1139 participants of the ABCD_1 study were selected, and 707 of them were included after the ABCD_2 re-evaluation. The participant selection flowchart is shown in Fig. 1. According to body weight change (cutoff, 5 kg), 87 participants were classified as G and 620 as NG. At the end of follow-up, 186 participants (26.3%) had body weight stable ( ± 1 kg). Body weight change distribution is reported in the Appendix (Supplemental Fig. A). The physical and clinical characteristics of the two groups are shown in Tables 1 and 2, and the dietary data are reported in Table 3. In particular, the reported prevalence of type 2 diabetes in at least one first or second-degree family member was not significantly different between G and NG (58.2% vs. 51.8%; *χ*^2^ = 2.99; *P* = 0.22). The two groups were comparable in terms of sex distribution and drug use; however, G were significantly younger than NG. Initial body weight and BMI were similar in both groups; nevertheless, 4 years later, G exhibited an average body weight gain of ~10 kg, while NG slightly but significantly decreased their body weight. The initial habitual energy intake was similar in both groups; however, 4 years later, the G group exhibited significantly higher habitual energy intake than the NG group. The correlation between body weight change and habitual energy intake is presented in the Appendix (Supplemental Fig. B). Dietary habits changed in both groups, as demonstrated by a reduction in the habitual intake of fats and an increase in carbohydrate intake. The MEDI-Lite score increased significantly in the NG group and remained unchanged in the G group. The physical activity level was not significantly different between G and NG (data not shown). The correlation between body weight change and HOMA-IR is presented in the Appendix (Supplemental Fig. C). The basal insulin serum concentrations and the HOMA-IR markedly increased in the G group, which also exhibited a higher prevalence of the alleles CG and GG (CG + GG, 56.1 vs. 43.0%; *P* < 0.05) of the PNAPL3 gene than the NG group. The change (Δ) in body weight was correlated with Δ insulin concentrations (*r* = 0.36; *P* < 0.001), Δ HOMA-IR (*r* = 0.32; *P* < 0.001), Δ MED-LITE score (*r* = −0.08; *P* < 0.05), Δ habitual energy intake (*r* = 0.17; *P* < 0.001), Δ glycemic index of diet (*r* = 0.08; *P* < 0.05), Δ glycemic load of diet (*r* = 0.14; *P* < 0.001), age (*r* = −0.19; *P* < 0.001). The multivariate logistic analysis demonstrated that becoming a body weight gainer (0 = not; 1 = yes) was independently associated with age, energy intake change (0 = $\le \,$ kcal/day; 1 = > 0 kcal/day) and carrying the CG or GG alleles of the PNAPL3 gene (0 = not; 1 = yes) (Table 4).

**Table 1:** Physical and clinical characteristics of the cohort divided according to a body weight change cutoff of 5 kg before and after 4 years.

	Non-gainers (*n* = 620)		*P*^a^	Gainers (*n* = 87)		*P*^a^
	Before	After		Before	After	
Males/females	231/389			32/55		
Age (years)	51 ± 13		–	44 ± 13^*^		
Offspring (*n*)	1.83 ± 1.17	1.86 ± 1.16	<0.001	1.46 ± 1.11^*^	1.52 ± 1.09^*^	<0.001
Hypertension (%)	30.2			22.0		
Use of (%):						
Beta-blockers	10.3	13.7		9.2	11.5	
Diuretics	10.6	13.1		5.7	7.9	
ACE/AR blockers	20.2	27.2		16.9	22.5	
Ca-channel blockers	5.1	6.6		2.2	2.2	
Statins	8.5	13.9		4.5	7.9	
Body weight (kg)	74.2 ± 14.7	72.9 ± 14.2	<0.001	76.7 ± 22.1	86.3 ± 23.7^*^	<0.001
Body mass index (kg/m^2^)	28.1 ± 5.1	27.6 ± 5.0	<0.001	27.8 ± 6.5	31.5 ± 7.2^*^	<0.001
Body circumferences (cm):						
Waist	95 ± 14	95 ± 12	0.96	95 ± 17	103 ± 15^*^	<0.001
Hip	105 ± 12	105 ± 9	0.09	105 ± 12	112 ± 13^*^	<0.001
Fat-free mass (kg)		51.5 ± 9.8	–		55.1 ± 11.8^*^	–
Fat mass (%)		28.8 ± 9.0	–		33.6 ± 9.2^*^	–
Blood pressure (mmHg):						
Systolic	127 ± 18	125 ± 17	<0.001	124 ± 20	121 ± 18	<0.001
Diastolic	80 ± 10	80 ± 10	0.36	82 ± 12	81 ± 10	0.18
Hart rate (beats/min)	69 ± 11	68 ± 11	<0.001	73 ± 12	72 ± 13	<0.05

Mean ± SD.

^a^Paired Student’s *t* test or *χ*^2^ when appropriate.

Unpaired Student’s *t* test: *P < 0.001.

**Table 2:** Biochemical and hormonal characteristics of the cohort divided according to a body weight change cutoff of 5 kg before and after 4 years.

	Non-gainers (*n* = 620)		*P*^a^	Gainers (*n* = 87)		*P*^a^
	Before	After		Before	After	
Blood concentrations of:						
Glucose (mg/dl)	89 ± 13	89 ± 11	0.17	87 ± 10	90 ± 11	< 0.05
Cholesterol (mg/dl)	216 ± 39	210 ± 39	<0.005	200 ± 36^**^	203 ± 37	0.97
HDL-c (mg/dl)	60 ± 15	62 ± 16	<0.001	59 ± 16	61 ± 18	0.69
eLDL-c (mg/dl)	136 ± 36	128 ± 36	<0.001	122 ± 32^**^	121 ± 32	0.39
Triglycerides (mg/dl)	103 ± 53	98 ± 54	<0.05	97 ± 59	104 ± 68	0.10
Uric acid (mg/dl)	5.0 ± 1.4	4.8 ± 1.3	<0.001	4.8 ± 1.3	5.0 ± 1.5	<0.05
Creatinine (mg/dl)	0.83 ± 0.20	0.82 ± 0.32	<0.001	0.81 ± 0.18	0.80 ± 0.17	0.69
Insulin (mcU/ml)	9.0 ± 4.6	9.2 ± 5.5	0.09	9.3 ± 5.1	12.9 ± 7.2^*^	<0.001
Δ Insulin		0.2 ± 3.4	–		3.6 ± 4.3^*^	–
HOMA-IR	2.10 ± 1.35	2.13 ± 1.47	0.12	2.07 ± 1.21	2.94 ± 1.75^*^	<0.001
HbA1c (%)	5.6 ± 0.4	5.4 ± 0.5	<0.001	5.6 ± 0.4	5.5 ± 0.4	0.08
eGFR (ml/min/1.73 m^2^)	92 ± 17	91 ± 17	0.82	98 ± 16	96 ± 16	<0.05
PNPLA3 rs738409 alleles (%)						
CC	57.0			43.8		
CG	36.4			40.4		
GG	6.6			15.7		<0.05
TM6SF2 rs58542926 alleles (%)						
CC	91.5			91.0		
CT	8.1			7.9		
TT	0.3			1.1		0.58

*eGFR* estimated glomerular filtration rate according to CKD-EPI, *eLDL-c* estimated low-density lipoprotein, *HbA1c* glycated hemoglobin, *HDL* high-density lipoprotein, *HOMA-IR* homeostatic model assessment of β-cell function and insulin resistance, *PNPLA3* patatin-like phospholipase domain containing 3, *TM6SF2* transmembrane 6 superfamily 2.

Mean ± SD.

^a^Paired Student’s *t* test or *χ*^2^ when appropriate.

Unpaired Student’s *t* test: *P < 0.001 and **P < 0.05 vs body weight non-gainers.

**Table 3:** Dietary characteristics of the cohort divided according to a body weight change cutoff of 5 kg before and after 4 years.

	Non-gainers (*n* = 620)		*P*^a^	Gainers (*n* = 87)		*P*^a^
	Before	After		Before	After	
Energy intake (kcal/day)	1434 ± 532	1325 ± 353	<0.001	1383 ± 337	1444 ± 392^*^	<0.001
Daily intake of:						
Carbohydrate, g	118 ± 30	166 ± 54	<0.001	112 ± 32	182 ± 58^*^	<0.001
%	33.5 ± 6.5	48.1 ± 9.5	<0.001	32.4 ± 7.1	48.3 ± 7.6	<0.001
Fats, g	71 ± 19	44 ± 16	<0.001	71 ± 21	48 ± 15	<0.001
%	46.7 ± 5.5	30.6 ± 5.9	<0.001	47.0 ± 6.3	31.3 ± 8.6	<0.001
Proteins, g	68 ± 17	65 ± 18	<0.001	70 ± 19	71 ± 18	0.68
%	20.0 ± 2.7	20.3 ± 3.5	0.09	20.8 ± 3.4	20.2 ± 2.8	0.14
MEDI-Lite score	9.9 ± 1.9	10.3 ± 2.1	<0.001	9.9 ± 1.9	9.9 ± 2.3^**^	0.84
Glycemic index	59 ± 4	58 ± 5	<0.001	58 ± 5	59 ± 5	0.56
Glucose load	65 ± 18	96 ± 32	<0.001	61 ± 19	106 ± 33^*^	<0.001

Mean ± SD.

^a^Paired Student’s *t* test.

Unpaired Student’s *t* test: ^*^P < 0.001 and ^**^P < 0.05 vs body weight non-gainers.

**Table 4:** Multivariate regression analysis of factor associated with the condition of body weight gainer (Δ body weight ≥5 kg from 2011 to 2015).

	Odds ratio	95% confidence interval		*P*
		Lower	Upper	
Age	1.031	1.022	1.040	<0.001
Δ Energy intake (0, ≤0; 1, >0 kcal/day)	2.257	1.345	3.789	<0.005
Δ Glucose load (0, ≤0; 1, >0)	0.811	0.345	1.910	0.63
Δ MEDI-LITE score (0, ≤0; 1, >0)	0.727	0.459	1.151	0.17
PNPLA3 CG,GG alleles (0, not; 1, yes)	1.700	1.066	2.709	<0.05

*Δ* change, *PNPLA3* patatin-like phospholipase domain containing 3.

The final HOMA-IR value was correlated with final values of age (*r* = 0.17; *P* < 0.001), sex (male = 0, female = 1; *r* = −0.11; *P* < 0.005), BMI (*r* = 0.49; *P* < 0.001), waist circumference (*r* = 0.51; *P* < 0.001), waist-to-hip ratio (*r* = 0.40; *P* < 0.001), FFM-kg (*r* = 0.28; *P* < 0.001), FM% (*r* = 0.30; *P* < 0.001), energy intake (*r* = 0.11; *P* < 0.005). No significant correlation was observed with MEDI-LITE score (*r* = −0.05, *P* = 0.17), PNPLA3 CG + GG alleles (0=not, 1=yes; *r* = 0.07, *P* = 0.09), glycemic index (*r* = −0.02, *P* = 0.62), and glucose load (*r* = 0.06, *P* = 0.10). Multiple stepwise regression analysis demonstrated that only the final BMI (*P* < 0.001), waist circumference (*P* < 0.001), and energy intake (*P* < 0.05) were independently correlated with HOMA-IR according to the following equation:

HOMA-IR = −3.13 + (0.067 × BMI) + (0.033 × waist circumference) + (0.0001 × energy intake), *R*^2^ = 0.31 (*P* < 0.001).

## Discussion

In this study, G comprised ~12% of the initial cohort; they experienced a significant increase in body weight, ~10 kg more than the NG. The G and NG groups had comparable initial body weights, BMI, body fat distributions (Table 1), and habitual energy intakes. However, as expected, 4 years later, G significantly increased their energy intake, which was higher than that of NG (Table 3), thus confirming that energy intake is the main critical factor influencing energy balance and body weight change. In general, participants in the ABCD study improved their dietary choices following initial, albeit generic, nutritional suggestions provided during the ABCD_1 study. Therefore, the macronutrient composition of the habitual diet was similar in both groups. Four years later, both groups significantly reduced their habitual fat intake and increased their carbohydrate intake (protein intake remained unchanged). However, only NG significantly reduced the glycemic index of the diet and improved adherence to the Mediterranean diet, as indicated by the MEDI-Lite score. These data also suggest that individualized prevention campaigns promoting healthy lifestyles are effective. By addressing clinical issues, providing strategies, and offering personalized suggestions, they can guide lifestyle changes in a nonintensive manner.

We previously investigated this cohort for variants in the PNPLA3 and TM6SF2 genes to identify people at a higher risk of liver steatosis (PNPLA3) and fibrosis (TM6SF2), a condition frequently associated with obesity, diabetes, and metabolic syndrome [25]. Interestingly, in this study, the G group had a significantly higher prevalence of the CG and GG variants of PNPLA3 gene than that of the NG group. Multivariate analysis revealed that the change in body weight, such as in G, was independently associated with age, changes in energy intake, and the presence of the CG or GG alleles of the PNPLA3 gene. Consistent with our findings, Cinque et al. [26] investigated liver steatosis in a small cohort of individuals during the COVID-19 lockdown and found that the PNPLA3 G allele was independently associated with body weight gain. However, to the best of our knowledge, this is the first report of an independent association between these alleles and body weight gain or obesity in a general population cohort. This association does not appear to be related to chance, as it may have a congruent biological explanation. Indeed, PNPLA3 is expressed in response to feeding and is involved in lipid autophagy (lipophagy), particularly in the liver, and to a lesser extent in adipose tissue, especially when there is an excess of dietary fatty acids [27–29]. Therefore, a defective protein may not ensure an efficient mechanism of autophagy that protects liver cells from excessive fat accumulation. Hence, we hypothesized that PNPLA3 might contribute to the destruction of a small but significant amount of dietary-derived fats introduced in excess, thus contributing to mitigating a positive energy balance and consequent body weight gain. If we postulate that autophagy mechanisms can eliminate as little as 5 g (or even less) of fatty acids daily, it could potentially prevent around 2.0–2.5 kg of body weight gain per year. This aligns with the amount of weight gained over 4 years by the participants in the G group of our study. However, it is essential to emphasize that this is merely a hypothesis that requires further investigation and remains speculative at this stage.

The relationship between insulin resistance (IR) and obesity has not yet been elucidated. However, our study clearly demonstrates that IR does not precede weight gain; rather, it is a consequence of weight gain. In fact, initially, the G and NG groups had the same anthropometric characteristics and did not have different values of both serum insulin concentrations and HOMA-IR. However, 4 years later, insulin levels as HOMA-IR increased significantly in the G group, which exhibited values higher than those in the NG group. As expected, the final HOMA-IR independently correlated with measures of adiposity (BMI) of central fat distribution, such as waist circumference, and, interestingly, with the amount of habitual energy intake, regardless of the type of macronutrient excess. Therefore, the higher the energy intake, the greater the IR, which supports the possibility that IR is a protective response in favor of the intracellular environment when exposed to an excess of energy substrate. If an excessive amount of glucose rapidly accumulates inside the cell, there is a risk of osmotic damage. Second, IR is a protective mechanism against oxidative damage caused by excessive nutrient and energy accumulation. Supporting this hypothesis, mitochondrial oxidative stress is known to occur due to intracellular energy substrate overflow [30]. Furthermore, a high glucose flux might force pathways, including glycogen synthesis, polyol, and hexosamine pathways, and the production of advanced glycation end-product precursors (AGEs), resulting in cellular damage [31–33]. Hoehn et al. [34] demonstrated in vitro and in a murine model that mitochondrial superoxide production in response to energy substrate overflow drives IR, as it is a sensor of cellular nutrient homeostasis. Therefore, IR protects the intracellular environment from oxidative stress, and mitochondrial superoxide may be a signal that drives a cellular response to dampen glucose uptake via the antagonism of GLUT4. One logical consequence is that there might be a need to reconsider the idea of pharmacologically eliminating IR. Attempting to remove this protective mechanism could have adverse effects and should be approached with caution. Similar to all compensatory mechanisms, persistent IR is expected to favor other well-known clinical problems of metabolic syndromes, such as type 2 diabetes and atherosclerosis. Therefore, the only possible rational approach to treat IR seems to be to reduce energy intake with diet, possibly using anti-obesity drugs that regulate food intake when diet alone fails.

This study had some important limitations. First, only PNPLA3 and TM6SF2 genes were evaluated, and other known genes involved in beta cell function and IR were not considered. However, these two genes were included in other investigations of liver steatosis in the ABCD study. This study did not aim to investigate the specific genetic causes of body weight gain and insulin resistance; however, we did not exclude the need for further in-depth genetic investigations of this cohort. Another important limitation is that we used HOMA-IR as a surrogate assessment of insulin resistance instead of the gold standard technique, that is, the euglycemic hyperinsulinemic clamp (EHC). However, EHC has been previously shown to correlate well with HOMA-IR values [35]; it is not a simple technique to perform in large cohorts, and to our knowledge, there is a paucity of studies using this technique beyond the first 6 months post weight loss bariatric surgery interventions [36, 37]. Unfortunately, we did not perform body composition analysis in the ABCD_1 study; therefore, we could not include body composition changes in our analysis. However, the evolution of body circumference was consistent with body weight change, and we are confident that the data on body composition modification would not have contradicted the conclusions of this study.

This study demonstrated that excessive energy intake is primarily associated with body weight gain and that genetic factors might favor a positive energy balance. Body weight gain is followed by insulin resistance, which is correlated with energy intake, and is probably a protective mechanism against intracellular oxidative stress resulting from energy substrate overflow.

### Supplementary information

Supplementary Figure A

Supplementary Figure B

Supplementary Figure C

## Data Availability

Individual de-identified participant data underlying the results reported in this article will be shared with researchers who provide a methodologically sound proposal. Data will be available beginning at 9 months and ending at 36 months after the publication of the article. Proposals should be directed toward silvio.buscemi@unipa.it. To gain access, the data requestors must sign a data-access agreement.
