# Supplementary figures and images for: Factors associated with body weight gain and insulin-resistance: a longitudinal study
Source: Nutr Diabetes. 2024 Apr 22;14:21. doi: 10.1038/s41387-024-00283-5 (PMC11035547; doi:10.1038/s41387-024-00283-5)

**Supplementary Figure A**

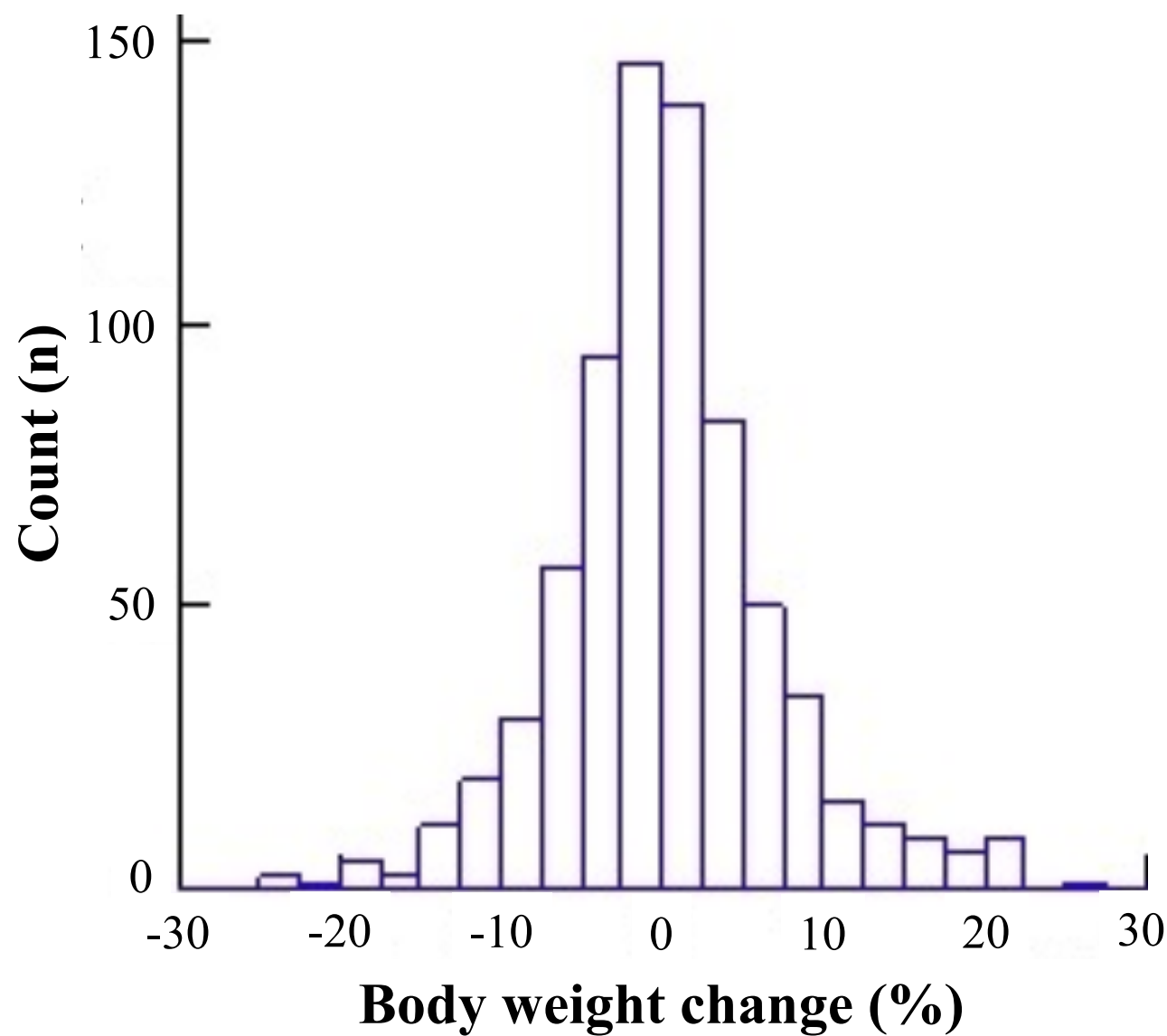

Weight change distribution in the cohort.

Supplement: Supplementary file 1 — Supplementary Figure A [file 41387_2024_283_MOESM1_ESM.pdf]
