# Supplementary material for: Factors associated with body weight gain and insulin-resistance: a longitudinal study
Source: Nutr Diabetes. 2024 Apr 22;14:21. doi: 10.1038/s41387-024-00283-5 (PMC11035547; doi:10.1038/s41387-024-00283-5)
Supplement: Supplementary file 3 — Supplementary Figure C [file 41387_2024_283_MOESM3_ESM.pdf]

Supplementary Figure C

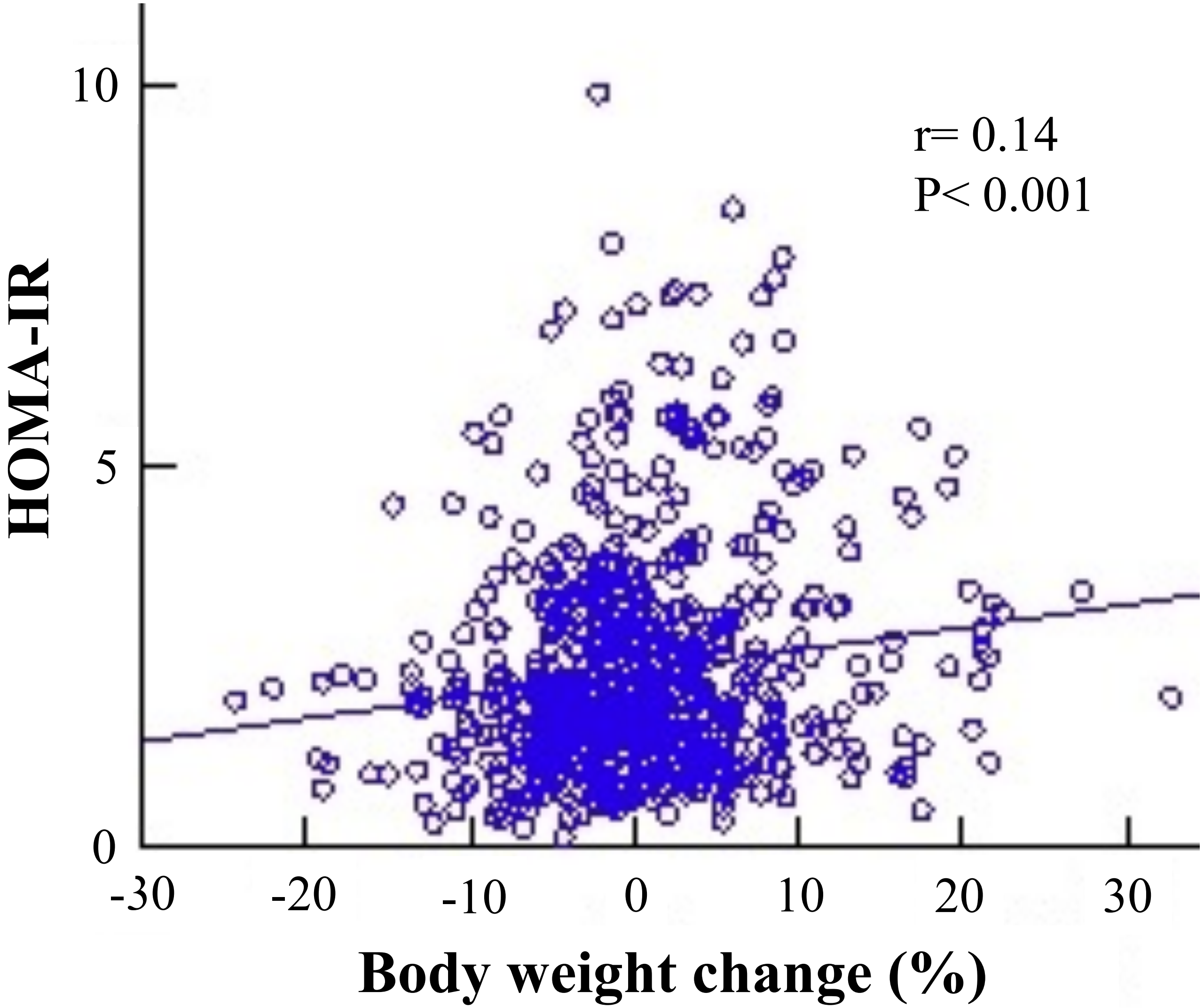

Correlation between 4-year change in body weight and HOMA-IR in the cohort.
